# Quantitative evaluation of tissue stiffness around lesion by sound touch elastography in the diagnosis of benign and malignant breast lesions

**DOI:** 10.1371/journal.pone.0219943

**Published:** 2019-07-18

**Authors:** Leidan Huang, Mengke Ma, Zhen Du, Zheng Liu, Xuehao Gong

**Affiliations:** 1 Department of Ultrasound, Xinqiao Hospital, Army Medical University, Chongqing, China; 2 Department of Ultrasound, First Affiliated Hospital of Shenzhen University, Second People’s Hospital of Shenzhen, Shenzhen, Guangdong, China; 3 Anhui Medical University, Hefei, Anhui, China; University of Montreal, CANADA

## Abstract

The purpose of our study is to assess the diagnostic performance of quantitative evaluation of tissue stiffness around lesion by Sound Touch Elastography (STE) in distinguishing between benign and malignant breast lesions. A total number of 160 breast lesions from 160 female patients were examined by STE. Resona 7 was equipped with “shell” function to measure elastic modulus values of tissue in the region of surrounding lesion quantitatively. The contours of the lesion were required to be delineated. The elastic modulus values of tissue in the region of 1mm, 2mm, and 3mm outside the boundary were acquired. The elastic modulus values included maximum elastic modulus (E_max_), mean elastic modulus (E_mean_), minimum elastic modulus (E_min_), and elastic modulus standard deviation (E_sd_). All lesions were confirmed by histopathology. We compared the differences of the above elastic modulus values between benign and malignant groups. Receiver operating characteristic (ROC) curve was drawn with the histological diagnostic results as the gold standard. Sensitivity and specificity were calculated to evaluate the diagnostic performance of STE. Operator consistency was also analyzed. Among the 160 lesions, 100 (62.5%) were benign and 60 (37.5%) were malignant. In the region of 1mm, 2mm, and 3mm surrounding the lesion, E_max_, E_mean_, and E_sd_ of malignant group were significantly higher than those of the benign group (all *P*<0.05). When the “shell” was 3mm, E_max_ had the highest AUROC value (AUROC = 0.998). Regarding the measurement of elastic modulus values, all the intra-class correlation coefficient (ICC) values of the inter-operator consistency were greater than 0.75 for E_max_, E_mean_, and E_sd_. Therefore, quantitative evaluation of tissue stiffness around lesion by STE has the potential to distinguish between benign and malignant breast lesions.

## Introduction

Breast cancer is one of the cancers that seriously threaten the health of women in the world [1]. Compared with other cancers, breast cancer has a better therapeutic effect and a lower recurrence rate [1]. Therefore, early detection and diagnosis are undoubtedly of great significance. In clinical practice, conventional ultrasound (US) is widely used for the diagnosis of benign and malignant breast lesions [2]. In comparison with other imaging methods, such as mammography and breast MRI, the conventional US has advantages of radiation-free, cost-effective, and real-time. However, conventional US showed low specificity in diagnosing breast lesions [2, 3].

With the development of ultrasound technology, elastography was put forward by Ophir to evaluate tissue stiffness in 1991 [4], and strain imaging was applied to differentiate malignant breast lesions from benign ones [5]. Benign breast lesions tend to be soft, whereas malignant lesions tend to be stiff. Strain imaging includes strain elastography (SE) and acoustic radiation force impulse imaging (ARFI) [6]. SE and ARFI are qualitative and semi-quantitative methods to evaluate tissue stiffness. Moreover, manual compression is required when using SE. SE is highly dependent on the operator thus its repeatability is not very high. In recent years, Sound Touch Elastography (STE) has been used as a novel elastography tool to measure tissue stiffness quantitatively [7]. Acoustic radiation force creates shear wave in soft tissue by external mechanical vibration. The propagation of shear wave and displacement of tissue were detected to calculate elastic modulus values. On Mindray (Mindray Medical International Ltd., Shenzhen, China) Resona 7 ultrasound system, this technology is called STE. Compared with SE, STE has the characteristic of simple operation and high repeatability because it does not require manual pressure from the operator. Compared with SE, STE could provide multiple elastic parameters, including Young’s modulus (E), shear modulus (G) and shear wave velocity (C) using the same ultrasound equipment. Currently, many studies have used shear wave elastography (SWE) to obtain stiffness information of tissue for differentiating benign and malignant breast lesions [8]. However, there is no universal standard to select the measurement area of breast lesion. Some studies selected the whole lesion as the measurement area to get elastic modulus values [9, 10], whereas some studies chose the stiffest part at internal or peripheral of the lesion to obtain elastic modulus values [11, 12].

Meanwhile, it is reported that not only the inside of malignant breast lesion is stiff but also the peritumoral tissue because breast carcinoma cell have the biological characteristic to infiltrate into peritumoral tissues [13, 14]. Zhou et al called this finding the “stiff rim” sign and reported that the “stiff rim” sign is valuable in the diagnosis of breast lesions [15]. However, previous studies have the limitation that they only judge whether the lesion presents the “stiff rim” sign qualitatively from color elastography map. The quantitative way to judge the presence of stiff rim sign has not yet been discussed. The aim of our study was to evaluate the diagnostic performance of quantitative measurement of tissue stiffness around lesion by STE in differentiating malignant breast lesions from the benign ones.

## Materials and methods

### Patients

Our retrospective study was agreed by the Institutional Review Board. Written consent forms were obtained from all participants for the scientific analysis of ultrasonic images and histopathologic results. From June 2017 to December 2017, 160 consecutive women (mean age, 45.7±12.3 years; age range 23–67 years) were participated in this study. The inclusion criteria of our study were as follows: (1) breast lesions were detected by conventional US or palpation; (2) breast lesions were solid or nearly solid (cystic composition <20%). The exclusion criteria of our study were as follows: (1) the women were pregnant or breast-feeding; (2) the women who had prosthesis implants in breast; (3) the size of breast lesions is larger than 30mm or less than 5mm; (4) STE image is incomplete; (5) histopathologic results were not obtained; (6) breast lesions were treated by any intervention, such as breast surgery, chemotherapy or radiotherapy. Finally, a total number of 160 breast lesions were included in this study.

### Image acquisition

Resona 7 ultrasound system (Mindray Medical International Ltd., Shenzhen, China) equipped with L11-3U linear array transducer (bandwith frequency of 3–11 MHz) was used to perform conventional US and STE. The conventional US and STE examinations were conducted by one of two operators with more than 2 years of experience in breast US and STE. During the examination, patients were kept in the supine position with breast and axilla fully exposed. Firstly, routine US examinations were performed in all patients to choose the breast lesion. In the case of multiple lesions in the same patient, the most suspicious lesion was selected to obtain transverse and longitudinal images. Gain and depth were adjusted to clearly show the lesion. Moderate ultrasonic coupling agents were smeared on the surface of breast. STE was performed by the same operator for the breast lesion. STE mode was switched at the longest length of breast lesions. The range of STE display scale was set as 0–140 kPa automatically for every lesion. In the region of interest (ROI), color map was used to demonstrate the stiffness of tissue: very soft tissue was colored by dark blue. With the increase of stiffness, the colors were light blue, green, yellow, orange, and red to represent the scale of stiffness from soft to hard.

The operator was required to avoid pressing and moving the transducer to minimize unintentional pre-compression. To weaken artificial stiffness, transducer was put on the surface of breast vertically as gently as possible. Patients were asked to hold their breath for several seconds to acquire stable STE images. Breast lesion was shown in the middle of screen. ROI box was adjusted to include surrounding tissue, comprising pectoral muscle, parenchyma, and subcutaneous fat. To ensure the reliability of STE, interfering factors such as obvious cystic parts or calcification were avoided when setting the ROI box on the image. In STE mode, both B-mode and STE images were simultaneously shown on a display screen. The grayscale B-mode image was displayed on the left hand side of the screen. Subtranslucent color STE image was superimposed on the corresponding B-mode image and the superimposed image was shown on the right hand side of the screen. The STE image of each lesion was recorded as a cine-loop and stored in the ultrasound instrument for subsequent analysis by the operator.

### Image evaluation

**Fig 1:**
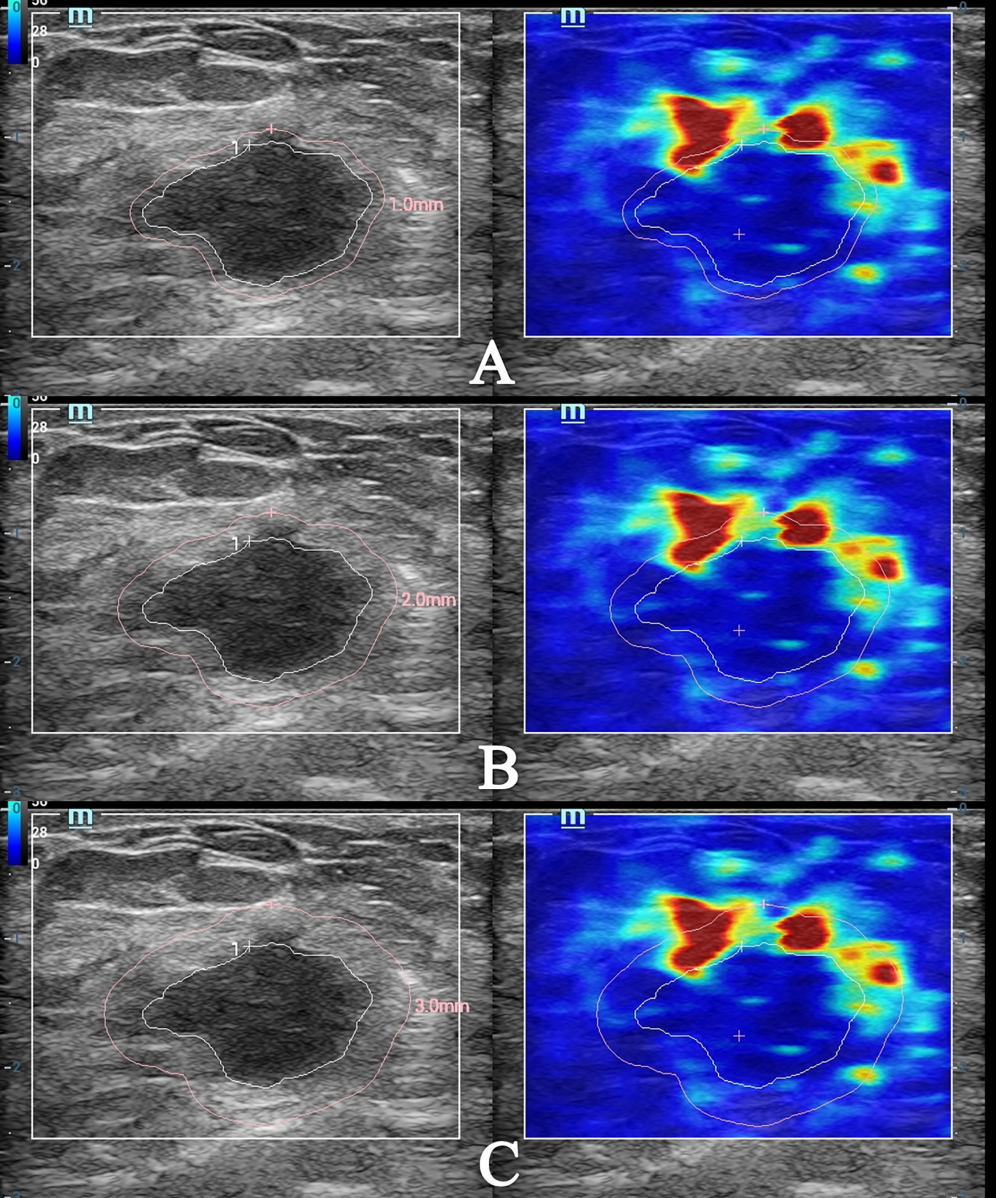
Images of the measurement of elastic modulus values by STE when the “shell” was 1mm, 2mm, and 3mm. (A) The E_max_, E_mean_, E_min_, and E_sd_ of tissue stiffness around the lesion were 257.50 kPa, 47.59 kPa, 4.60 kPa, and 44.79 kPa respectively when the “shell” was 1mm; (B) The E_max_, E_mean_, E_min_, and E_sd_ of tissue stiffness around the lesion were 276.32 kPa, 53.03 kPa, 4.03 kPa, and 51.05 kPa respectively when the “shell” was 2mm; (C) The E_max_, E_mean_, E_min_, and E_sd_ of tissue stiffness around the lesion were 276.32 kPa, 52.81 kPa, 4.03 kPa, and 51.16 kPa respectively when the “shell” was 3mm.

The stored video was retrieved to select three frames of static image for quantitative analysis of tissue stiffness around the lesion. The images were chosen when the breast lesion and its surrounding tissues showed a steady and even color. The contours of the lesion were traced and delineated by sliding trackball on the grayscale B-mode image. Next, the “shell” function key on the control panel was pressed. For the first time, the “shell” was adjusted to 1mm. The elastic modulus values of tissue in the region of 1mm outside the boundary could be obtained. The ultrasound system automatically calculated the elastic modulus values in the “shell” area, including maximum elastic modulus (E_max_), mean elastic modulus (E_mean_), minimum elastic modulus (E_min_), and elastic modulus standard deviation (E_sd_). The above measurement steps were repeated three times in order to calculate the average value. The “shell” was adjusted to 2mm and 3mm for the second and third time to acquire the above elastic modulus values of tissue in the region of 2mm and 3mm outside the boundary, respectively (Fig 1). The operator was not informed of the clinical and pathological information of patients before image evaluation.

### Inter-operator consistency of STE

The other operator repeated the same steps of the previous operator to obtain E_max_, E_mean_, E_min_, and E_sd_. The STE operation process was independently performed by two operators. In addition, the operation process was guaranteed not to interfere with each other. The transducer was removed and reapplied between each acquisition by the two operators in the same lesion.

### Histopathological diagnosis

Histopathology was considered as the gold standard in diagnosis of breast lesion. All pathological tissue specimens were obtained from surgical resection or ultrasound-guided needle biopsy. The final histopathological diagnosis was made by a pathologist with more than 20 years of experience in breast diseases who had no knowledge of the STE results.

### Statistical analysis

The continuous data included the age of patients, maximum diameter of lesion, and elastic modulus values of lesion. Shapiro-Wilk test was used to verify whether the data was normally distributed. Data of normal distribution was represented by mean ± standard deviation. Data which was not normally distributed was represented by median and interquartile range (IQR). Differences of elastic modulus values between benign and malignant breast lesions were compared by Mann-Whitney U test for independent samples. Receiver operating characteristic (ROC) curve proposed by DeLong et al. was drawn to acquire area under the receiver operating characteristic curve (AUROC), sensitivity, and specificity by using all the data set [16]. Then, the optimal cutoff values were determined by Youden index (maximum of sensitivity + specificity– 1). Intra-class correlation coefficient (ICC) was used to evaluate the inter-operator consistency of STE. The range of ICC is from 0 to 1. ICC was interpreted as follows [17]: 0–0.39, poor reliability; 0.40–0.59, fair reliability; 0.60–0.74, good reliability; 0.75–1.00, excellent reliability. Statistical analysis was performed by the SPSS 23.0 software (SPSS, Chicago, IL) and MedCalc 15.8 software (MedCalc Software, Mariakerke, Belgium). The distribution of E_max_, E_mean_, E_min_, and E_sd_ was drawn by GraphPad Prism 7.0 software (GraphPad Software, Inc., San Diego). According to the Bonferroni correction, a *P* value less than 0.05/12 = 0.0042 was considered statistically significant.

## Results

Among the 160 lesions, 100 (62.5%) of them were diagnosed as benign and the other 60 (37.5%) lesions were diagnosed as malignant by histopathology. The pathological diagnosis results of benign and malignant lesions are shown in Table 1. The age of patients in the benign group were 30.52 ± 11.05 (range = 21–49) years old. The age of patients in the malignant group were 49.85 ± 13.55 (range = 37–67) years old. The mean age for the malignant group was higher than that of the benign group (*P* = 0.000). The maximum lesion’s diameter for the benign group was 18.63 ± 6.97mm (range = 12.1mm–25.4mm). The maximum lesion’s diameter for the malignant group was 16.54 ± 5.83mm (range = 11.8mm–26.3mm). There was no significant difference in the maximum diameter of lesion between the benign and the malignant groups (*P* = 0.176).

**Table 1:** The histopathological results of 160 breast lesions.

Histopathological results	No.
Benign	100
Fibroadenoma	32
Fibrocystic change	16
Fibroadenomatous hyperplasia	11
Mastitis	11
Intraductal papilloma	11
Ductal epithelial hyperplasia	10
Adenosis	7
Benign phyllodes tumor	2
Malignant	60
Invasive ductal carcinoma	53
Invasive ductal carcinoma with intraductal carcinoma in situ	5
Intraductal carcinoma in situ	1
Papillary carcinoma	1

When the “shell” was 1mm, 2mm, and 3mm, the E_max_, E_mean_, and E_sd_ of malignant group were significantly higher than that of benign group (*P* = 0.000 for all) (Table 2, Table 3 and Table 4). The E_min_ of malignant group was higher than that of benign group without significant difference when the “shell” was 1mm (*P* = 0.587) and 3mm (*P* = 0.985). When the “shell” was 2mm, the E_min_ of malignant group was lower than that of benign group, but the difference was not statistically significant (*P* = 0.819).

**Table 2:** The elastic modulus values of benign and malignant lesions when the “shell” was 1mm*.

Parameters	All lesions (n = 160)	Benign lesions (n = 100)	Malignant lesions (n = 60)	*P* value
E_max_ (kPa)	76.05 (51.85–133.05)	58.53 (41.89–73.50)	165.97 (120.83–206.18)	0.000
E_mean_ (kPa)	24.65 (18.09–33.89)	20.11 (14.82–24.89)	36.03 (28.71–44.57)	0.000
E_min_ (kPa)	4.73 (3.19–7.43)	4.73 (3.50–7.29)	4.75 (2.44–7.59)	0.587
E_sd_ (kPa)	14.31 (8.24–22.30)	10.51 (7.11–14.31)	26.12 (20.77–30.52)	0.000

*The values obtained over operator 1

**Table 3:** The elastic modulus values of benign and malignant lesions when the “shell” was 2mm*.

Parameters	All lesions (n = 160)	Benign lesions (n = 100)	Malignant lesions (n = 60)	*P* value
E_max_ (kPa)	84.36 (55.95–168.91)	61.75 (45.08–79.74)	184.03 (155.89–225.15)	0.000
E_mean_ (kPa)	24.48 (17.99–37.37)	19.91 (14.35–24.29)	39.91 (33.14–47.12)	0.000
E_min_ (kPa)	4.02 (2.80–6.47)	4.02 (3.13–6.10)	3.91 (1.71–7.29)	0.819
E_sd_ (kPa)	15.23 (9.08–27.43)	10.80 (7.14–14.52)	30.68 (25.38–34.05)	0.000

*The values obtained over operator 1

**Table 4:** The elastic modulus values of benign and malignant lesions when the “shell” was 3mm*.

Parameters	All lesions (n = 160)	Benign lesions (n = 100)	Malignant lesions (n = 60)	*P* value
E_max_ (kPa)	86.12 (57.16–174.68)	64.50 (46.50–83.67)	206.02 (168.62–228.70)	0.000
E_mean_ (kPa)	23.21 (16.90–38.03)	19.02 (13.78–22.86)	40.85 (33.50–45.78)	0.000
E_min_ (kPa)	3.41 (1.66–5.21)	3.36 (1.95–5.05)	3.50 (1.42–6.46)	0.985
E_sd_ (kPa)	14.55 (9.10–27.39)	10.38 (7.08–13.92)	30.61 (26.57–34.01)	0.000

*The values obtained over operator 1

**Fig 2:**
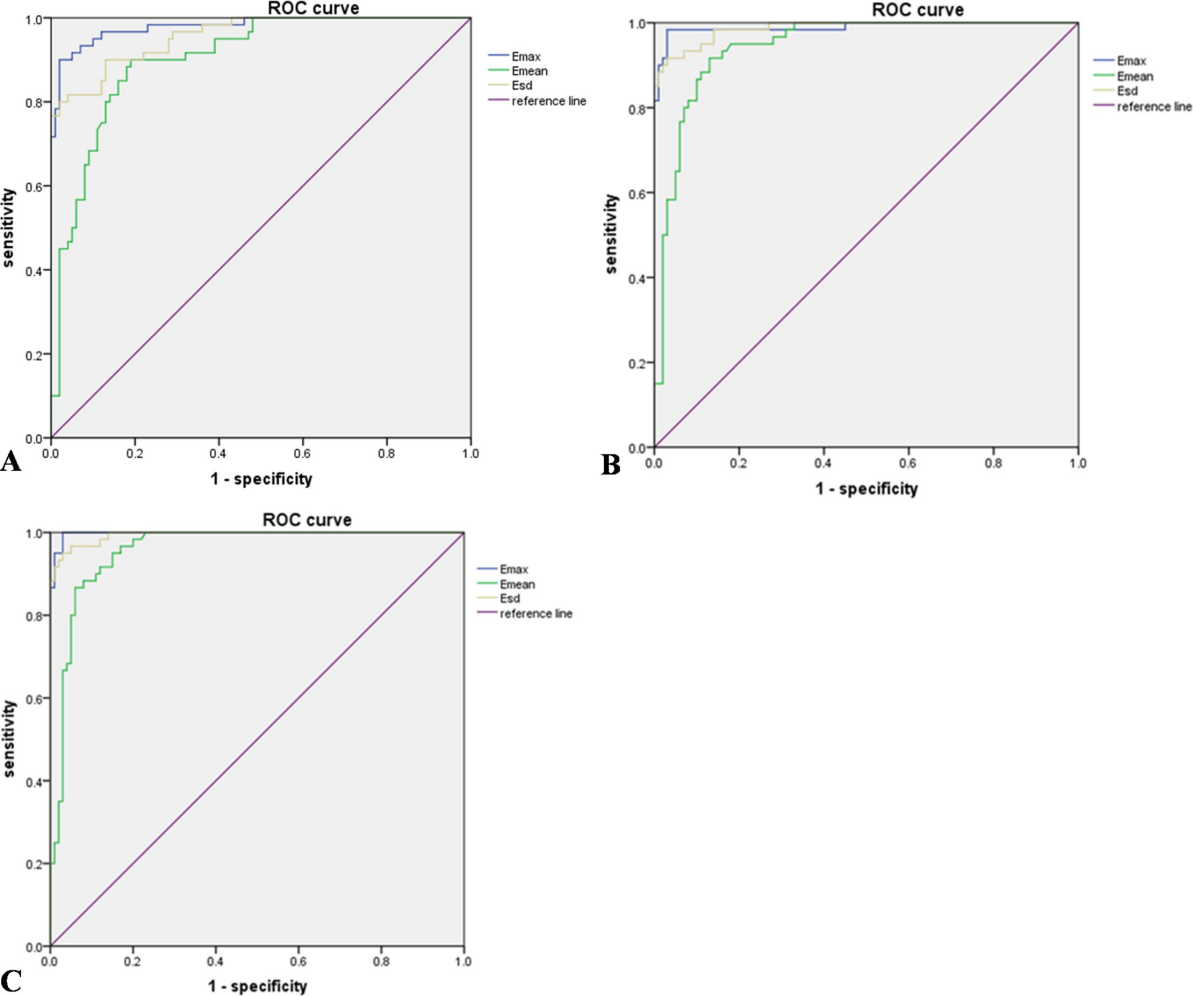
The ROC curves of E_max_, E_mean_, and Esd (A, The ROC curves of E_max_, E_mean_, and E_sd_ when the “shell” was 1mm. B, The ROC curves of E_max_, E_mean_, and E_sd_ when the “shell” was 2mm. C, The ROC curves of E_max_, E_mean_, and E_sd_ when the “shell” was 3mm.).

**Fig 3:**
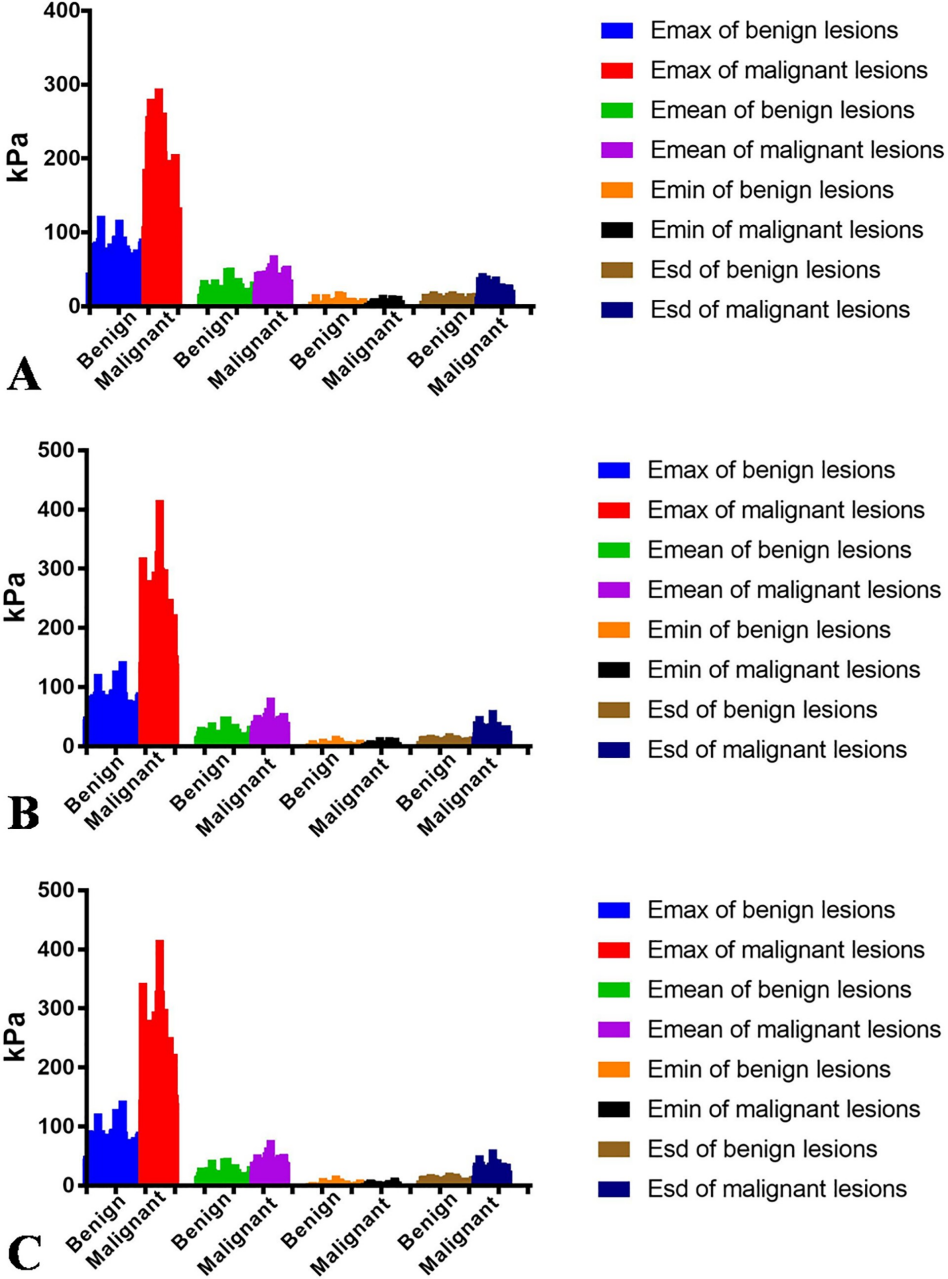
Distribution of E_max_, E_mean_, E_min_, and E_sd_ between benign and malignant breast lesions (A, Distribution of E_max_, E_mean_, E_min_, and E_sd_ when the “shell” was 1mm. B, Distribution of E_max_, E_mean_, E_min_, and E_sd_ when the “shell” was 2mm. C, Distribution of E_max_, E_mean_, E_min_, and E_sd_ when the “shell” was 3mm.).

According to the ROC curve analysis (Fig 2A), when the “shell” was 1mm, the optimal cutoff values of E_max_, E_mean_, and E_sd_ were 99.20 kPa, 25.87 kPa, and 19.09 kPa respectively, and the AUROC were 0.980, 0.904, and 0.957 respectively. When the “shell” was 2mm, the optimal cutoff values of E_max_, E_mean_, and E_sd_ were 100.43 kPa, 27.89 kPa, and 18.39 kPa respectively according to the ROC curve analysis (Fig 2B), and the AUROC were 0.989, 0.944, and 0.987 respectively. According to the ROC curve analysis (Fig 2C), when the “shell” was 3mm, the optimal cutoff values of E_max_, E_mean_, and E_sd_ were 100.43 kPa, 31.22 kPa, and 18.33 kPa respectively, and the AUROC were 0.998, 0.957, and 0.994 respectively. Regardless of whether the “shell” was 1mm, 2mm, and 3mm, the E_max_ had the largest AUROC among other elastic modulus values (*P*<0.05). When the shell was 3mm, its AUROC is the largest. The details are shown in Table 5. All the intra-class correlation coefficient (ICC) of the inter-operator consistency was greater than 0.75 for E_max_, E_mean_, and E_sd_. The inter-operator consistency between two separate measurements of the same lesion by different operators had good reliability for the measurement of E_max_, E_mean_, and E_sd_ (Table 6). As for E_min_, the reliability was moderate. The distribution of E_max_, E_mean_, E_min_, and E_sd_ between benign and malignant breast lesions is shown in Fig 3 in detail.

**Table 5:** Diagnostic performance of STE in differentiating malignant and benign lesions*.

Shell	Cutoff value (kPa)	Sensitivity (%)	Specificity (%)	AUROC (95% CI)
1mm				
E_max_	99.20	90.0	98.0	0.980 (0.961, 0.999)
E_mean_	25.87	90.0	81.0	0.904 (0.858, 0.950)
E_sd_	19.09	80.0	98.0	0.957 (0.929, 0.985)
2mm				
E_max_	100.43	98.3	97.0	0.989 (0.974, 1.000)
E_mean_	27.89	91.7	87.0	0.944 (0.911, 0.978)
E_sd_	18.39	91.7	97.0	0.987 (0.974, 1.000)
3mm				
E_max_	100.43	98.6	97.0	0.998 (0.994, 1.000)
E_mean_	31.22	86.7	94.0	0.957 (0.928, 0.987)
E_sd_	18.33	95.0	97.0	0.994 (0.986, 1.000)

*The values obtained over operator 1

**Table 6:** The Inter-operator consistency of elastic modulus values by STE.

Parameters	ICC		
	“shell” was 1mm	“shell” was 2mm	“shell” was 3mm
E_max_	0.785 (0.717–0.838)	0.874 (0.832–0.906)	0.888 (0.850–0.917)
E_mean_	0.865 (0.820–0.899)	0.908 (0.876–0.931)	0.930 (0.905–0.948)
E_min_	0.629 (0.526–0.715)	0.628 (0.524–0.713)	0.594 (0.484–0.686)
E_sd_	0.825 (0.768–0.869)	0.889 (0.851–0.918)	0.914 (0.884–0.936)

## Discussion

The BI-RADS classification has high sensitivity but relatively low specificity [18]. In practice, some benign breast lesions were misdiagnosed as malignant lesions, which resulted in unnecessary biopsy. The reason for the relatively low specificity of BI-RADS US reporting may be that the category of breast lesion was determined by characteristics of two-dimensional grayscale ultrasonography [19]. The characteristics of malignant lesions are as follows: taller-than-wide shape, fuzzy contours, irregular margin, hypoechogenicity or marked hypoechogenicity, and microcalcifications [20]. However, the two-dimensional grayscale ultrasonography of malignant and benign breast lesions have overlaps to some extent. With the improvement of ultrasonic equipment and technology, especially the application of elastic imaging technology, the diagnosis of malignant and benign breast lesions by ultrasound has made great progress [5].

Elastic imaging technology imitates palpation. It is highly dependent on the differences in the biomechanical properties of tissues. However, elastic imaging technology has higher sensitivity and accuracy than traditional palpation [5]. Qualitative and semi-quantitative assessment of tissue stiffness could be achieved. Generally, the five-point method proposed by Itoh is adopted in qualitative assessment [21]. With an optimal cutoff value between three and four points, its sensitivity, specificity, and AUROC are 86.5%, 89.8%, and 0.919 respectively [21]. However, the five-point method proposed by Itoh is highly dependent on operator’s experience. The sensitivity, specificity, and AUROC of the semi-quantitative assessment are 92.4%, 91.1%, and 0.944 respectively when the best cutoff value for strain ratio is 3.05 [5]. As for the quantitative assessment, different elastic modulus values have different diagnostic performance. It is reported that the ranges of optimal cutoff value between malignant and benign breast lesions with a 2-mm ROI for elastic modulus values are as follows: 33.3–80.0 kPa for E_mean_; 46.7–93.8 kPa for E_max_; 6.3–13.9 kPa for E_sd_ [8]. In literature, the sensitivity, specificity, and AUROC of different elastic modulus values are as follows: 88.6%, 89.9%, and 0.932 for E_mean_; 90.3%, 81.8%, and 0.931 for E_max_; 89.2%, 91.1%, and 0.899 for E_sd_ [8].

Elastography has promising diagnostic performance in differentiating malignant and benign breast lesions, which has been confirmed by many studies [5]. It is a popular research direction in the field of identifying malignant and benign breast lesions. Many scholars dedicate effort to the in-depth study of elastography. Evans and Tozaki et al. reported that the stiffest part of malignant breast lesion appears in the periphery of the lesion rather than inside [13, 14]. This feature on the color elastography map is defined as the “stiff rim” sign [15]. In this study, STE was used to measure the stiffness of tissue around lesion quantitatively with the combination of novel “shell” function. The elastic modulus values of tissue in the region of 1mm, 2mm, and 3mm within the surroundings of lesion were compared, including E_max_, E_mean_, E_min_, and E_sd_. The results showed that the E_max_, E_mean_, E_min_, and E_sd_ of malignant lesions were higher than those in benign lesions when the “shell” was 1mm, 2mm, and 3mm. In a previous study, Evans obtained four elastic modulus values by SWE to diagnose malignant breast lesion, namely E_max_, E_mean_, E_min_, and E_sd_ [13]. During the measurement, ROI was placed in the stiffest part inside or around the lesion [13]. The results of our study are similar to that. Evans et al. took a circular ROI with a diameter of 1mm according to the color elastography map [13]. They first looked at the color elastography map to choose a relatively stiff area. Then the ROI was placed to obtain a series of elastic modulus values. It was difficult to measure the elastic modulus values of surrounding tissue of the lesion as a whole. In our study, we sampled the whole annular region of tissue around the lesion, rather than a portion of the surrounding tissue. The assessment method of stiff rim sign is more informative. Although Evans and Tozaki et al. reported that the stiff rim sign was found around malignant breast lesions, their studies did not analyze the diagnostic performance of stiff rim sign in the differentiation of malignant and benign lesions [13, 14]. Our study not only compared the difference of elastic modulus values between benign and malignant breast lesions, but also evaluated the diagnostic efficiency of them.

In the present study, the E_max_, E_mean_, and E_sd_ of malignant breast are significantly much higher than those of benign lesions. Our result is consistent with what previous studies have shown [9, 13]. Zhou et al. analyzed the diagnostic performance of stiff rim sign by SWE in differentiating between benign and malignant breast lesions. They found that the sensitivity, specificity, and AUROC of stiff rim sign are 94.6%, 89.1%, and 0.918 respectively [15]. However, in the study of Zhou et al., if the breast lesions do not have stiff rim sign when the SWE display scale was first set at 180 kPa, the display scale will be turned down to such a level that a portion of peritumoral tissue is coded in stiff color [15]. In our study, the display scale of color elastography map was set at a fixed range uniformly for every lesion, which made the acquisition of STE much easier. When the “shell” was 1mm, the ICC for E_max_, E_mean_, and E_sd_ were 0.785, 0.865, and 0.825 respectively. When the “shell” was 2mm, the ICC for E_max_, E_mean_, and E_sd_ were 0.874, 0.908, and 0.889 respectively. When the “shell” was 3mm, the ICC for E_max_, E_mean_, and E_sd_ were 0.888, 0.930, and 0.914 respectively. According to our statistical analysis results, the inter-operator consistency of STE is good, which is consistent with other studies [9, 15, 22].

Several studies confirmed that E_max_ showed better diagnostic efficiency in differentiating malignant and benign breast lesions in comparison with E_mean_ and E_sd_ [15, 22, 23]. Our results also showed that the diagnostic performance of E_max_ is better than that of E_mean_ and E_sd_, whether the “shell” was 1mm, 2mm, and 3mm. When the “shell” was 1mm, the sensitivity, specificity, and AUROC of E_max_ were 90.0%, 98.0%, and 0.980 respectively. When the “shell” was 2mm, the sensitivity, specificity, and AUROC of E_max_ were 98.3%, 97.0%, and 0.989 respectively. When the “shell” was 3mm, the sensitivity, specificity, and AUROC of E_max_ were 98.6%, 97.0%, and 0.998 respectively. However, Xu et al. found that E_sd_ had the highest diagnostic value in comparison with E_max_ and E_mean_ [9]. Considering the reasons for the different results, we recognized that the possibility may be as follows: E_max_ shows the maximum stiffness of the stiffest part of the lesion in ROI. E_mean_ indicates the average value of tissue stiffness in ROI, which is related to the size of ROI as well as the maximum and minimum values of elastic modulus of tissue in the ROI. E_sd_ is the standard deviation of elastic modulus value, which reflects the consistency and heterogeneity of stiffness distribution in the ROI. If the stiffness of tissue in the ROI is more consistent, the E_sd_ is lower. If the stiffness of tissue in the ROI is more heterogeneous, the E_sd_ is higher. Nevertheless, the higher E_sd_ do not mean the greater likelihood of malignancy for breast lesions. This is because some malignant lesions have uniform increased stiffness areas at the periphery, while some malignant lesions are surrounded by only local increased stiffness regions. When the “shell” was 3mm, the diagnostic performance of E_max_ was much better in comparison with the circumstance when the “shell” was 1mm and 2mm. The border of malignant breast lesions tends to be ill-defined. When the “shell” was 1mm and 2mm, manually delineating the contours of lesion may include a portion of the lesion itself in the ROI. The “stiff rim” sign was confirmed to offer significant diagnostic performance not only for solid breast lesions, but also for non-mass-like breast lesions.

Several previous studies indicated two main explanations for the presence of “stiff rim” sign. First, the malignant breast lesions tend to infiltrate into surrounding tissues [13, 21, 24, 25]. The dynamic changes of infiltration of cancer cells into peripheral interstitial tissues and a desmoplastic reaction result in the formation of a mixed band, which may consist of cell proliferation, lymphocyte infiltration, fibrosis, and tumor angiogenesis. Second, the intrinsic tissue of malignant breast lesions is hard, which exceeds the shear wave threshold [14, 26]. The energy of shear wave is attenuated and absorbed in the peritumoral region of malignant breast lesions. The ultrasonic system misinterprets the shear wave with poor waveform and low amplitude inside the lesion as a low-speed shear wave. Instead, the speed of shear wave is correctly reflected in the vicinity of malignant breast lesions.

To our knowledge, there was no published research focusing on STE to evaluate the presence or absence of “stiff rim” sign. Besides, it was also the first time to take the “shell” function to measure the elastic modulus values of whole region around breast lesion. Finally, our present study has several limitations. First, the breast lesions included in this study were confirmed by histopathology, which may lead to selection bias. Second, the size of the sample was relatively small. In addition, the pathological types of malignant breast lesions were relatively insufficient in our study. Most of malignant lesions were invasive ductal carcinoma. The optimal cutoff value between different pathological types of breast carcinoma was not explored. Third, in this research, the “shell” was only selected as 1mm, 2mm, and 3mm to explore the diagnostic performance of elastic modulus values. If we further refine the “shell”, such as 1.5mm and 2.5mm, the result may be unknown. In addition, we did not combine STE and BI-RADS category to differentiate benign and malignant breast lesions. In the future work, we intend to improve the above limitations as much as we can.

## Conclusions

In conclusion, STE was of great value in the diagnosis of malignant and benign breast lesions by quantitatively measuring elastic modulus values of tissue around the lesion. The E_max_, E_mean_, and E_sd_ of peripheral tissues of malignant lesions were significantly higher than those of benign lesions. Among the above elastic modulus values, E_max_ was the best parameter, especially when the “shell” was 3mm. This method of quantitative evaluation of the presence or absence of “stiff rim” sign for breast lesion is simple and feasible. It not only has high sensitivity and specificity, but also has good consistency among observers. STE could be a valuable tool to diagnose malignant and benign breast lesions. Large samples and multi-center cooperation are needed for further studies.

## Supporting information

S1 Data Dataset of breast lesions. (XLSX)
